# PARP inhibition increases sensitivity to cisplatin in non-small-cell lung carcinoma via the induction of TET-dependent hydroxymethylation

**DOI:** 10.3389/fcell.2025.1677261

**Published:** 2025-10-24

**Authors:** Nevena Grdović, Anja Tolić, Mirna Jovanović, Jovana Rajić, Marija Đorđević, Marijana Stojanović, Stefan Marković Hadžić, Tijana Marković, Ana Sarić, Svetlana Dinić, Jelena Arambašić Jovanović, Mirjana Mihailović, Ana Podolski-Renić, Milica Pešić, Melita Vidaković, Aleksandra Uskoković

**Affiliations:** ^1^ Department of Molecular Biology, Institute for Biological Research “Siniša Stanković”, National Institute of the Republic of Serbia, University of Belgrade, Belgrade, Serbia; ^2^ Department of Neurobiology, Institute for Biological Research “Siniša Stanković”, National Institute of the Republic of Serbia, University of Belgrade, Belgrade, Serbia

**Keywords:** non-small-cell lung carcinoma cells, chemosensitivity, PARP inhibitor, niraparib, 5hmC, ten–eleven translocations enzymes

## Abstract

**Introduction:**

Platinum-based chemopotentiation by poly (ADP-ribose) polymerase (PARP) inhibitors (PARPi) has been confirmed in some non-small-cell lung carcinoma (NSCLC) models, but the molecular mechanisms of PARPi synergy with chemotherapeutics remain poorly clarified. This study aimed to evaluate the efficacy of PARPi niraparib in mitigating resistance to cisplatin through increased ten-eleven translocation (TET) enzymatic activity and 5-hydroxymethylcytosine (5hmC) level in human NSCLC model.

**Methods:**

The chemosensitivity of human NSCLC, sensitive and multidrug-resistant (NCI-H460 and NCI-H460/R, respectively), lung adenocarcinoma cell line (A549), and normal embryonic lung fibroblasts (MRC-5) to increasing concentrations of cisplatin was studied by the MTT assay. The MTT and the median effect analyses were used to evaluate the efficacy of niraparib combinatorial synergy with cisplatin, dimethyloxalylglycine (DMOG), and vitamin C. The inhibition of PARP activity was determined by the PARP activity assay. The global level of 5hmC was examined by confocal microscopy, slot-blot, and flow cytometry, which was used for monitoring cell death at different stages. The protein level of lamin B, PARP1, procaspase-3, and TETs was assessed by Western blotting. The expression of the TET enzyme was analyzed by real-time quantitative PCR.

**Results:**

The chemosensitivity to cisplatin was found to be correlated with the level of 5hmC in NSCLC and MRC-5 cell lines. A549 was characterized with the lowest sensitivity to cisplatin and the 5hmC level and was selected for chemosensitization via PARPi-dependent restoration of the 5hmC level. Treatment of A549 cells with niraparib resulted in decreased PARP activity, increased 5hmC level, and synergism with cisplatin by promoting cisplatin-induced apoptosis. The treatment with niraparib and cisplatin resulted in synergistic effect in the hydroxylase activity of TET2 in terms of increased 5hmC production in A549 cells.

**Conclusion:**

The improved response to cisplatin following PARPi-mediated sensitization of A549 cells, accompanied by the restoration of 5hmC levels, provides additional insights into the epigenetic mechanisms underlying the synergy between PARPi and cisplatin. The use of PARPi in clinical settings could increase the number of patients enriched in 5hmC, who might benefit from platinum-based therapy. Additionally, targeting epigenetic marks could provide a molecular basis for personalized cancer therapy directed to overcome tumor resistance.

## 1 Introduction

Lung cancer is the leading cause of cancer-related mortality worldwide, with the predominant non-small-cell lung carcinoma (NSCLC) type accounting for almost 85% of the overall lung cancers (Guo et al., 2022). Platinum compounds are the foundation of chemotherapy treatments for NSCLC, with cisplatin being the most frequently used one (Szupryczyński et al., 2024). However, the limited clinical effects resulting from primary and acquired resistance to platinum agents impose the need for improving the responsiveness of cancer cells by sensitizing NSCLC cells to cisplatin action.

The concept of using poly (ADP-ribose) polymerase (PARP) inhibitors (PARPi) in the treatment of lung cancer has been developing in recent years. The hypothesis that the combined effects of PARP inhibition with platinum compounds may improve platinum-based NSCLC responsiveness has been postulated, but the molecular mechanisms underlying this synergy have not been completely deciphered, at least not in the broad spectrum of NSCLC cells (Cheng et al., 2013; Stolzenburg et al., 2022). Platinum-based agents exert their cytotoxic effects by forming DNA adducts that disrupt DNA structure and inhibit cell division, ultimately resulting in cell death. Consequently, the ability of cancer cells to repair DNA damage has been recognized as a key mechanism in mediating platinum resistance (Cheng et al., 2013). Accordingly, the mechanism aimed at overcoming chemoresistance should rely on preventing DNA repair, which will increase the likelihood of effective treatment. One of the clarified mechanisms for the increased sensitivity of lung cancer cells to platinum agents by PARP inhibition is based on synthetic lethality effects produced by the simultaneous impairment of PARP1 and PARP2, which is involved in base excision repair, with dysfunction of genes associated with DNA damage response such as homologous recombination (HR). Thus, disabling of cells from repairing DNA leads to selective death of cancer cells with an impaired HR/DNA repair system. Beyond the well-established synthetic lethality of PARPi in HR-deficient cells, emerging evidence suggests that PARP inhibition may also exert therapeutic effects in HR-proficient NSCLC (Jiang et al., 2019; Oza et al., 2015).

With growing evidence that DNA methylation plays an important role in the acquisition of platinum resistance, the inhibition of DNA methyltransferase (DNMT) activity emerged as a potential therapeutic strategy to overcome tumor resistance, which was supported by findings from clinical trials (Wang et al., 2023; Singh et al., 2022). In recent years, along with DNMT inhibitors, the ten–eleven translocation (TET) enzymes, a family of DNA demethylases, were found to be important for overcoming drug resistance due to the DNA hypomethylating outcome. The TET enzymes (which belong to Fe^2+^/α-ketoglutarate-dependent oxygenases) initiate active DNA demethylation by catalyzing the sequential oxidation of 5-methylcytosine (5mC) to 5-hydroxymethylcytosine (5hmC) and then to 5-formylcytosine (5fC) and 5-carboxylcytosine (5caC), with the 5hmC being the most stable form and independent epigenetic marker *per se* (Bachman et al., 2014). Decreased expression of TET proteins and lower 5hmC levels are hallmarks of many cancer types, including lung cancer (Rasmussen and Helin, 2016; Yang et al., 2013). It is considered that the repression of TET1 in lung cancer could be at least partially responsible for silencing of tumor suppressor genes, tumor growth, and resistance (Forloni et al., 2016). Given that global loss of 5hmC, resulting from aberrant TET enzymatic activity, is associated with decreased responsiveness to platinum-based chemotherapy, 5hmC may serve both as a global epigenetic marker for assessing chemotherapeutic response and as a potential target for resensitizing platinum-resistant tumors. Indeed, increasing TET activity with the concomitant rescue of 5hmC loss was already suggested as a potential targetable pathway for enhancing the chemosensitivity of platinum-resistant tumors (Tucker et al., 2018). Higher levels of 5hmC will provide a positive signal for inhibition of the proliferation of cancer cells and will also provide a better response to cancer therapy (Bisht et al., 2022). Therefore, the regulation of TET activity has direct implications in cancer biology, particularly in the context of drug resistance. Interactions of TET enzymes with other proteins as well as post-translational modifications of TETs are among the most prominent mechanisms that regulate the activity of TET enzymes (Joshi et al., 2022). Accordingly, the interacting proteins and post-translational modifications of TETs may be important not only for achieving but also for changing the net effects of TET-mediated cytosine oxidation and DNA hydroxy/(de)methylation pattern.

Our previous results clearly showed that PARP-dependent PARylation, a covalent posttranslational modification of proteins with poly (ADP-ribose) (PAR) polymers, inhibits TET hydroxylase activity, while the increase in DNA hydroxymethylation upon the inhibition of PARylation has been unequivocally demonstrated (Tolić et al., 2022). Hence, the stimulation of TET-dependent DNA hydroxymethylation by inhibited PARylation could be exploited as valuable approach for PARPi’ potential in the treatment of cancers characterized by loss of 5hmC, with the aim of increasing the sensitivity to cisplatin. This study aimed to elucidate whether PARP inhibition could sensitize NSCLC cells to cisplatin via the induction of TET activity. These insights could ascribe the a aspect of the role of PARPi in sensitizing cancer cells to cisplatin, which could lead to targeted therapy of patients with low 5hmC levels and a chemoresistant phenotype.

## 2 Materials and methods

### 2.1 Cell lines

Human NSCLC (NCI-H460) and lung adenocarcinoma (A549) cell lines were obtained from American Type Culture Collection (ATCC, Rockville, MD, USA). Multidrug-resistant NCI-H460/R cells were selected from NCI-H460 cells by continuous exposure to stepwise increasing concentrations of doxorubicin (Pesic et al., 2006). Normal embryonic lung fibroblasts (MRC-5) were purchased from the European Collection of Authenticated Cell Cultures (ECACC, Port Down, UK). All cells were maintained at 37 °C in a humidified 5% CO_2_ atmosphere. The NSCLC cell lines, both sensitive and resistant, were grown in RPMI-1640 medium (Capricorn Scientific GmbH, Ebsdorfergrund, Germany), supplemented with 10% fetal bovine serum (FBS), 2 mM L-glutamine, 4.5 g/L glucose, 10,000 U/mL penicillin, 10 mg/mL streptomycin, and 25 μg/mL amphotericin B solution. A549 and MRC-5 were grown in Dulbecco’s modified Eagle medium (DMEM, Capricorn Scientific GmbH, Ebsdorfergrund, Germany) supplemented with 10% FBS, 2 mM L-glutamine, 5,000 U/mL penicillin, and 5 μg/mL streptomycin.

### 2.2 MTT assay

The MTT assay was used to measure the metabolic activity of the cells, which indirectly measures the viability of cells. Cells grown in 75-cm^2^ tissue flasks were trypsinized, seeded into flat-bottomed adherent 96-well cell culture plates in an appropriate medium (2,000 cells/well for NCI-H460, NCI-H460/R, and A549 and 5,000 cells/well for MRC-5), and incubated overnight. The cells were treated with increasing concentrations of cisplatin (1 μM–25 μM) for 72 h. Additionally, A549 cells were exposed to treatment with niraparib (1 μM–25 μM) for 72 h. The combined effects of cisplatin with niraparib, along with niraparib with dimethyloxalylglycine (DMOG) and vitamin C, were studied in A549 cells. In simultaneous treatments that lasted 72 h, three concentrations of niraparib (0.5, 1, and 1.5 μM) were combined with five concentrations of cisplatin (0.25 μM–5 μM), while DMOG (10, 25, and 50 μM) and vitamin C (50, 100, and 250 μM) were combined with five increasing concentrations of niraparib (0.25 μM–5 μM). At the end of the treatment period, a medium containing 0.2 mg/mL MTT was added to each well. After incubation (3 h, 37 °C, and 5% CO_2_), the MTT-containing medium was removed, and 200 μL of dimethyl sulfoxide (DMSO) was immediately added to each sample. The absorbance was measured at 570 nm on a Multiskan Sky Microplate Spectrophotometer (Thermo Fisher Scientific, USA). The half-maximal inhibitory concentration (IC_50_) of each compound was calculated using Graph Pad Prism 6.0 (GraphPad Software, Inc., USA).

### 2.3 Median effect analysis

The median effect analyses are based on the median effect principle established by Chou and Talalay (1984), which was used to calculate the combination index (CI) value for the interaction between niraparib and cisplatin, niraparib and DMOG, and niraparib and vitamin C. The analyses were carried out using CalcuSyn software (Biosoft, Cambridge, UK). We used at least three data points for each single drug in each designed experiment. The non-constant ratio combination was chosen to determine the effect of both drugs in combination. CI < 1 indicates synergism, CI = 1 indicates an additive effect, and CI > 1 indicates antagonism.

### 2.4 Drug interaction assessment

SynergyFinder+ is an advanced online tool designed to evaluate drug–drug interactions and detect potential synergistic effects. We applied this tool to analyze the interaction between niraparib and cisplatin using dose–response data obtained from MTT assays performed on A549 cells for various drug combinations. SynergyFinder+ utilized multiple models to estimate synergies between the drugs. The zero interaction potency (ZIP) model improves the analysis by comparing the drugs’ dose–response curves. It assumes that there is no interaction between the drugs and calculates their expected effect. Any deviations from this expected effect are interpreted as a synergy (Duarte and Vale, 2022). Next, the Loewe additivity model is based on the concept that two drugs in combination behave like a single drug administered at different doses. This model allows the assessment of whether the combined doses yield an additive effect or demonstrate synergism. Additionally, the highest single agent (HSA) model compares the effects of the drug combination to the maximum effects produced by the single drugs. A combination that exceeds this maximal single-agent effect indicates a synergistic interaction. Finally, the Bliss independence model assumes that the two drugs act through independent mechanisms. It estimates the expected effect based on the probability of unrelated events occurring simultaneously. If the observed effects of the combination exceed this expected value, it indicates a synergistic interaction. For all four models, synergy is reflected by a positive score above 0 (Supplementary S1 SynergyFinder report).

### 2.5 Slot-blot assay

Genomic DNA from NCI-H460, NCI-H460/R, A549, and MRC-5 was isolated by the Bio-On-Magnetic-Beads (BOMB) magnetic bead-based protocol (Oberacker et al., 2019). Isolated DNA was diluted to a final concentration of 10 ng/μL in Tris-EDTA buffer and denatured in 0.4 M NaOH and 10 mM EDTA at 95 °C for 10 min, followed by immediate cooling on ice. The reaction was neutralized by ice-cold ammonium acetate (pH 7.0) at a final concentration of 1 M. All samples were kept on ice until they were loaded to a Hybond N+ positively charged nylon membrane using a vacuum-driven slot-blot system Bio-Dot (Bio-Rad), as per the manufacturer’s instructions. The membrane was then dried at 80 °C for 30 min in a drying oven before further processing. Each DNA sample was loaded in duplicate, allowing the membrane to be cut in half—one half was used for immunoblotting of 5hmC, and the other was stained with 0.1% methylene blue in 0.5 M sodium acetate (pH 5.2). For immunoblotting, membranes were blocked in 5% milk for 1 h at room temperature and then incubated with rabbit anti-5hmC antibody (1:10,000; Active Motif, Carlsbad, CA) at 4 °C overnight. The following day, the membranes were incubated with HRP-conjugated anti-rabbit secondary antibody (1:20,000; Abcam) for 1 h at room temperature. Detection was performed using the iBright FL1500 Imaging System. The relative 5hmC levels were determined by densitometry using TotalLab ver. 1.10 electrophoresis software (Phoretix International Ltd, Newcastle upon Tyne, UK) with normalization to the methylene blue-stained bands.

### 2.6 Immunocytochemical detection of the global 5hmC level

NCI-H460, NCI-H460/R, A549, and MRC-5 cell lines were cultured in wells of an 8-well chamber slide (seeded 20,000 cells per well) under standard conditions for 24 h. On the next day, the cells were gently washed with PBS and subsequently fixed in 2% paraformaldehyde for 10 min at room temperature. Next, cell membranes were permeabilized using 0.25% Triton X-100 for 10 min at room temperature. DNA denaturation was carried out by treating the cells with 2 N HCl at 37 °C for 30 min. After rinsing, nonspecific binding was blocked using 3% bovine serum albumin (BSA). The cells were then incubated with a rabbit anti-5hmC antibody (1:10,000; Active Motif, Carlsbad CA), followed by incubation with the secondary antibody donkey anti-rabbit IgG conjugated to Alexa Fluor 555 (1:400; Invitrogen, Carlsbad, CA). After thorough washing with PBST and water, the slide was overlaid with Mowiol mounting medium and covered with a glass coverslip. Fluorescence imaging was performed by a Leica TCS SP5 II confocal microscope using a 543-nm laser for excitation and a ×63 magnification.

### 2.7 PARP activity assay

PARP activity was determined in nuclear lysates of A549 cells treated with niraparib (1.5 µM and 10 µM) using a commercial colorimetric PARP Assay Kit (Trevigen, Gaithersburg, MD, USA) according to the manufacturer’s instructions and expressed as units of PARP1 activity.

### 2.8 Flow cytometric detection of 5hmC

A549 cells used for the assessment of 5hmC DNA content were cultivated in 6-well plates (100,000 cells/well) and treated with 1.5 µM niraparib, 5 µM cisplatin, or 1.5 µM niraparib +5 µM cisplatin for 72 h. Cell harvesting included removal of the cultivation medium, detaching the cells (0.1% trypsin-EDTA in HBSS, at 37 C for 4 min), and collection of detached cells in DMEM supplemented with 10% FBS, 1% glutamine, and 1% penicillin/streptomycin. After centrifugation (500 x g, 5 min), cells were resuspended in cold PBS, and an aliquot containing 0.5 x 10^6^ cells was taken for further processing. After pelleting (500 x g, 5 min), cells were subjected to fixation and permeabilization as follows: a) the initial fixation/permeabilization by incubation in BD Cytofix/Cytoperm™ solution (BD Bioscience, San Jose, CA, USA) for 20 min at room temperature, b) additional permeabilization by incubation in BD Cytoperm Permeabilization Buffer Plus (BD Bioscience, San Jose, CA, USA) for 10 min on ice, and c) refixation by incubation in BD Cytofix/Cytoperm™ solution (BD Bioscience, San Jose, CA, USA) for 5 min on ice. Each fixation/permeabilization step was followed by a washing step with BD Perm/Wash Buffer (BD Bioscience, San Jose, CA, USA) prepared in accordance with the manufacturer’s instructions. Cells were further subjected to DNAse treatment aiming to expose 5hmC. Cells were resuspended in DNAse I (Sigma-Aldrich, St. Louis, MO, USA)-containing solution (100 μg/mL DNAse I in PBS supplemented with 2% BSA, 1 mM CaCl_2_, 0.5 mM MgCl_2_) and incubated at 37 ℃ for 1 h with continuous shaking. Staining of cells included the following steps: blocking of unspecific binding with 10% donkey serum at 4 ℃ for 30 min, incubation with rabbit anti-5hmC antibody (1:1,000; Active Motif, Carlsbad, CA) at 4℃ overnight, and incubation with donkey anti-rabbit IgG antibody conjugated with Alexa Fluor™ 488 (1:40; Invitrogen, Carlsbad, CA) at 4 ℃ for 30 min in the dark. After washing, stained cells were resuspended in BD Perm/Wash Buffer and analyzed on a CytoFLEX Flow Cytometer, V-B-R series (Beckman Coulter Life Sciences, Brea, CA, USA), using CytExpert software v2.4.

### 2.9 Cell apoptosis assay

Apoptosis of specifically treated A549 cells was assessed by Annexin V (Invitrogen, Carlsbad CA) and PI (Fluka, Buchs, Switzerland) staining. Cells were cultivated in 6-well plates (100,000 cells/well) and treated with 1.5 µM niraparib, 5 µM cisplatin, or 1.5 µM niraparib +5 µM cisplatin for 72 h with an additional 24-h-long incubation in DMEM supplemented with 10% FBS, 1% glutamine, and 1% penicillin/streptavidin upon completion of the specific treatment. Upon harvesting and washing in cold PBS, cells were resuspended in annexin V-binding buffer (10 mM HEPES, 150 mM NaCl, 2.5 mM CaCl_2_, pH 7.4) to 1 x 10^6^ cells/mL, and 100 µL was taken for staining. PI (1 µL of 100 μg/mL PI/annexin V-binding buffer) and annexin V conjugated with Alexa Fluor 488 (5 µL) were added into the aliquoted cell suspension and incubated for 15 min on ice in the dark. After the incubation period, 400 µL of annexin-binding buffer were added, and samples were analyzed without delay. Flow cytometric analysis was performed on a CytoFLEX Flow Cytometer, V-B-R series (Beckman Coulter Life Sciences, Brea, CA, USA), using CytExpert software v2.4.

### 2.10 Western blotting

A549 cells—control and treated with 1.5 µM niraparib, 5 µM cisplatin, or 1.5 µM niraparib +5 µM cisplatin—were lysed in lysis buffer containing 50 mM Tris HCl at pH 8.0, 150 mM NaCl, 1% Triton X100, and supplemented with Mix G cocktail of protease inhibitors (1X) at 4 ℃ for 30 min with sporadic vortexing. Equal amounts of cell lysates per group (30 µg) were separated by 12% sodium dodecyl sulfate polyacrylamide gel electrophoresis and transferred onto polyvinylidene difluoride membranes (Amersham Hybond P 0.45 PVDF, GE Healthcare Life Sciences). All primary antibodies were incubated at 4 ℃ overnight, followed by incubation with the appropriate horseradish peroxidase-conjugated secondary antibody for 60 min at room temperature. Antibodies against TET1 (09-872) and TET2 (#ABE364) were purchased from Merck Millipore, while antibodies against PARP1 (#sc-7150), lamin B (#sc-6217), and caspase-3 (#sc-7148) were purchased from Santa Cruz Biotechnology. Antibody against β-tubulin was raised against bovine brain β-tubulin (dr. Ursula Euteneuer). Signals were detected by using the enhanced chemiluminescence detection system according to the manufacturer’s instructions (Amersham Pharmacia Biotech, Amersham, UK). Membranes were stripped and reprobed with anti-β-tubulin antibody as a loading control. Relative protein levels were quantified by densitometry using TotalLab ver. 1.10 electrophoresis software (Phoretix International Ltd, Newcastle, upon Tyne, UK), with normalization to the amount of β-tubulin present in each sample.

### 2.11 Real-time quantitative PCR (RT-qPCR)

The 1 µg of total RNA extracted from A549 cells—control and treated with 1.5 µM niraparib, 5 µM cisplatin, or 1.5 µM niraparib +5 µM cisplatin—was treated with DNAse I and subjected to complementary DNA synthesis with a RevertAid First-Strand cDNA Synthesis Kit (Thermo Fisher Scientific, Waltham, MA, USA) using mixed oligo (dT) and random hexamer primers (1:1). The Maxima SYBR Green/ROX qPCR Master Mix (Thermo Fisher Scientific, Waltham, MA, USA) and the Quant Studio 3 Real-Time PCR system (Applied Biosystems, Carlsbad, CA, USA) were used for quantification of the examined mRNA. The thermal cycles included the following steps: an initial denaturation step at 95 ℃/10 min and 45 cycles of two-step PCR at 95 ℃/15 s and 60 ℃/60 s. The relative expression level of the analyzed genes was calculated by the comparative 2^−ΔΔCt^ method after normalization using GAPDH as an endogenous control. The primers were designed in Primer-BLAST (https://www.ncbi.nlm.nih.gov/tools/primer-blast/) for human sequences stored in GenBank. Statistical tests were performed on log2-transformed data. For the graphs, the mean values and error bars were back-transformed to the linear scale. The following primers were used:

hTET1-F AGAGACTGCCAACATTGCCT.

hTET1-R ATGATTTCCCTGACAGCAGCA.

hTET2-F AACATTCAGCAGCACACCCT.

hTET2-R TGCCCTCAACATGGTTGGTT.

hTET3-F ATCCGGGAACTCATGGAGGA.

hTET3-R CTGGTAGAGGGTGTCTCCGA.

hGAPDH-F CGGAGTCAACGGATTTGGTCGTAT.

hGAPDH-R AGCCTTCTCCATGGTGGTGAAGAC.

### 2.12 Statistical analysis

A statistical analysis of experimental data was performed using GraphPad Prism 6.0 (GraphPad Software, Inc., USA). The experiments were performed in triplicate, and data were expressed as the mean ± standard deviation (mean ± SD). One-way ANOVA followed by Tukey’s test was used for comparisons between groups, while two-way ANOVA followed by Dunnett’s or Tukey’s multiple comparisons tests were applied for synergism/antagonism and apoptosis studies. The difference was considered to be significant with a p-value ≤0.05 (*), ≤0.01 (**), ≤0.001 (***), and ≤0.0001 (****). The results of the statistical analysis and p-values are provided within Supplementary Material (Excel file S2).

## 3 Results

### 3.1 Chemosensitivity to cisplatin correlates with 5hmC level in NSCLC cell lines

**FIGURE 1:**
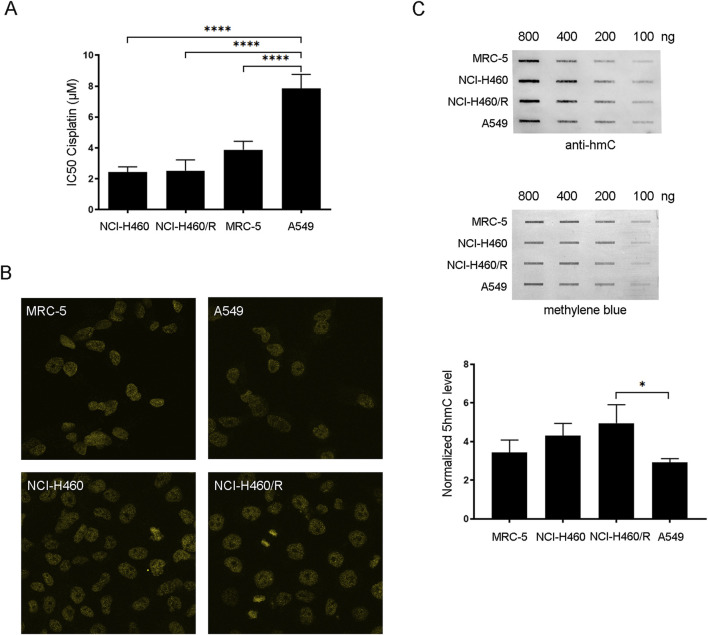
Chemosensitivity to cisplatin and the level of 5hmC in NSCLC (human NCI-H460 considered parental/sensitive cells, NCI-H460/R resistant to doxorubicin and other unrelated drugs that are substrates for the ABCB1 transporter and A549)) and normal fetal lung fibroblast cell lines (MRC-5). **(A)** IC_50_ values of cisplatin calculated from MTT cell viability assay performed after 72 h of treatment of NCI-H460, NCI-H460/R, A549, and MRC-5 with increasing concentrations of cisplatin. **(B)** Global levels of 5hmC in NCI-H460, NCI-H460/R, A549, and MRC-5 cells examined by confocal fluorescence microscopy using anti-5hmC antibody. **(C)** 5hmC level in genomic DNAs extracted from NCI-H460, NCI-H460/R, A549, and MRC-5 cells examined by slot-blot analysis. Slot-blot analysis was performed with anti-5hmC antibody on 100, 200, 400, and 800 ng of genomic DNA. Intensity of 5hmC bands was quantified and normalized against methylene blue staining in 800-ng samples. The results are presented as the means ± SD. Statistically significant differences between groups are indicated with * with a p-value ≤0.05 (*), ≤0.01 (**), ≤0.001 (***), and ≤0.0001 (****).

In order to identify candidate NSCLC cell lines suitable for cisplatin chemosensitization via PARPi-dependent restoration of 5hmC, chemosensitivity to cisplatin and the levels of 5hmC were determined in three human NSCLC cell lines (NCI-H460, NCI-H460/R, and A549) and normal human fetal lung fibroblast cells (MRC-5) (Figure 1). According to the literature, the NSCLC cell line A549 is more resistant to cisplatin compared to NCI-H460 cells (Chen et al., 2010; Zhang et al., 2025). In addition, NCI-H460/R cells, resistant to multiple drugs such as doxorubicin, etoposide, vinorelbine, and paclitaxel, show less collateral sensitivity to cisplatin compared to their parental NCI-H460 counterpart (Dinić et al., 2023). Chemosensitivity of NCI-H460, NCI-H460/R, A549, and MRC-5 cell lines to increasing concentrations of cisplatin was studied by the MTT assay. The IC_50_ values for cisplatin following 72 h of treatment with concentrations of cisplatin ranging from 0.5 to 8 μM are presented in Figure 1A. The comparative analysis of the IC_50_ values of cisplatin between NCI-H460, NCI-H460/R, A549, and MRC-5 cell lines showed that A549 exhibits the highest level of cisplatin resistance among the examined cell lines (p < 0.0001 for A549 vs NCI-H460, NCI-H460/R, and MRC-5) (Figure 1A). Namely, the IC_50_ value of cisplatin in A549 was 7.84 ± 0.92 µM, while in NCI-H460 and NCI-H460/R, the IC_50_ values were similar and estimated to be 2.44 ± 0.34 and 2.53 ± 0.70 µM, respectively. In MRC-5, the IC_50_ value was determined to be 3.89 ± 0.54 µM. The whole-cell level of 5hmC in NSCLC (NCI-H460, NCI-H460/R, and A549) and MRC-5 cell lines was examined by confocal fluorescence microscopy and slot-blot analysis, as a second inclusion criteria according to which the low 5hmC level should be a targetable pathway to be rescued by PARPi. Confocal fluorescence microscopy of 5hmC-stained NSCLC (NCI-H460, NCI-H460/R, and A549) and MRC-5 cells revealed that A549 cells exhibited the lowest level of DNA hydroxymethylation, with only minor 5hmC level observed (Figure 1B). For more accurate comparison, the slot-blot assay was conducted to examine the 5hmC level in genomic DNAs extracted from NSCLC (NCI-H460, NCI-H460/R, and A549) and MRC-5 cells (Figure 1C). The intensity of 5hmC bands were quantified and normalized against methylene blue staining. Slot-blot quantification of 800 ng of DNA isolated from tested cell lines confirmed that the A549 cell line is characterized by the lowest level of 5hmC, which is significantly reduced (1.69 fold) compared to the NCI-H460/R cells (p = 0.0239). The findings from our previous experiments indicated that the NCI-H460/R cells showed slight sensitivity to cisplatin when compared to the parental NCI-H460 cell line (Dinić et al., 2023), which aligns with the elevated levels of 5hmC detected in the resistant NCI-H460/R cells. Considering that the level of 5hmC was correlated with chemosensitivity, the A549 cell line was selected for further experiments, which were set up to test the possibility that increasing the 5hmC levels by inhibiting PARP activity with niraparib, a highly selective inhibitor of PARP1/2, will affect its resistance to cisplatin.

### 3.2 PARP inhibition affects 5hmC formation in the A549 cell line

**FIGURE 2:**
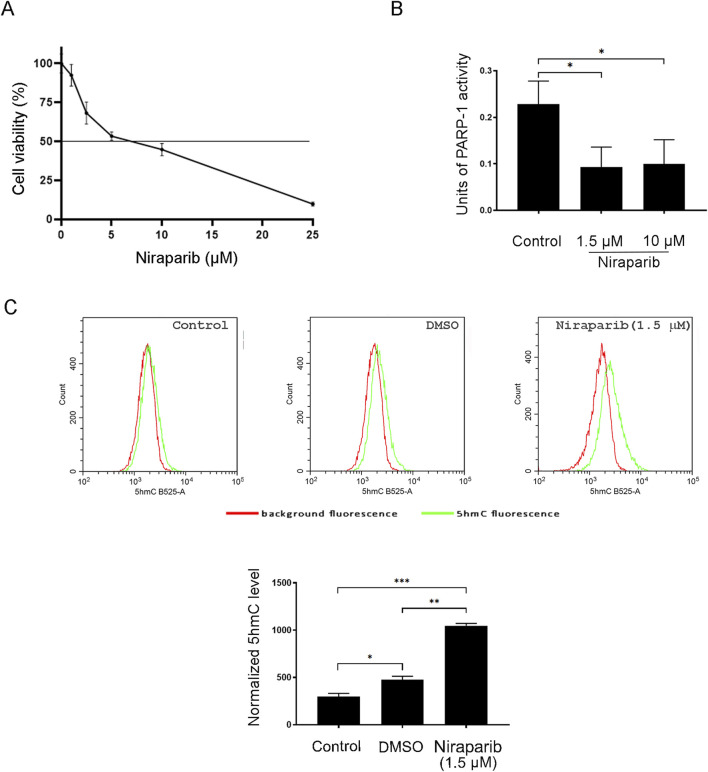
Effect of PARP inhibition on the 5hmC level in the A549 NSCLC cell line. **(A)** Cell viability assay of A549 cells after treatment with increasing concentrations of PARP inhibitor niraparib for 72 h **(B)** PARP activity assay performed with cell lysates from control and A549 cells treated with 1.5 and 10 µM niraparib. **(C)** Flow cytometric analysis of the control and A549 cells treated with DMSO (vehicle) and 1.5 µM niraparib, labeled with anti-5hmC antibody. The results of quantification are presented as the means ± SD. Statistically significant differences between groups are indicated with * with a p-value ≤0.05 (*), ≤0.01 (**), ≤0.001 (***), and ≤0.0001 (****).

Before investigating the effect of PARP inhibition on 5hmC production in A549 cells, the cytotoxicity of niraparib was assessed by MTT assay. According to the MTT analysis, the cytotoxic effect of niraparib was observed as a dose-dependent reduction in cells’ viability compared to the untreated control (Figure 2A). A 10 µM concentration of niraparib that we used in our previous work demonstrating the stimulating effect of PARPi on DNA hydroxymethylation (Tolić et al., 2022) markedly suppressed the A549 cell’s viability, with the reduction detected in ˃50% cells (Figure 2A). To select a more tolerable concentration of PARPi for potentially achieving the chemosensitization effect, we evaluated whether niraparib administered at a concentration of 1.5 µM, rendering more than 80% cells as viable, could effectively impede PARP activity. The level of PARP activity in cell lysates obtained from A549 cells, untreated and treated with 10 μM and 1.5 µM concentrations of niraparib, was determined by ELISA-based PARP activity assay (Figure 2B). Both concentrations of niraparib, 10 µM and 1.5 µM, significantly reduced PARP activity to almost the same extent of 2.30-fold (p = 0.0397) and 2.45-fold (p = 0.0321), respectively, compared to untreated A549 cells. Following that, cells treated with 1.5 µM niraparib were subjected to flow cytometric analysis for the assessment of 5hmC levels (Figure 2C). The mean fluorescence intensity (MFI) recorded for A549 cells treated with 1.5 µM niraparib was higher than the MFI in controls (p = 0.0005) and DMSO (p = 0.0012).

### 3.3 PARP inhibitor niraparib enhanced the susceptibility of A549 cells to cisplatin-induced apoptosis

**FIGURE 3:**
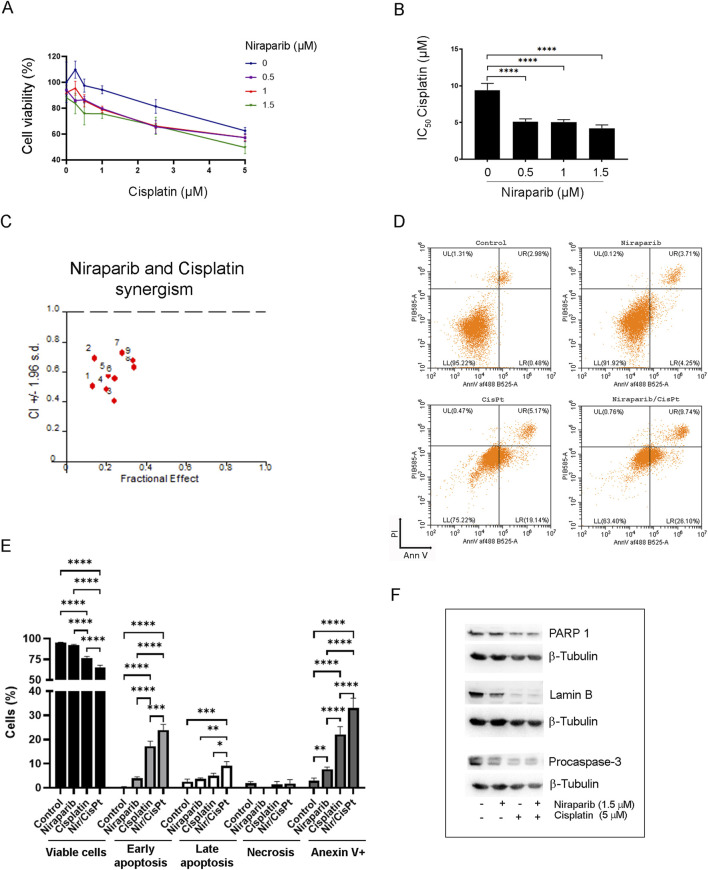
Effect of niraparib on the susceptibility of A549 cells to cisplatin-induced apoptosis. **(A)** Cell viability assay of A549 cells after combined treatment of increasing concentrations of niraparib and cisplatin. **(B)** IC_50_ values of cisplatin for the control and A549 cells treated with 0.5, 1, and 1.5 µM niraparib. **(C)** Interactions between niraparib (0.5, 1, and 1.5 μM) and cisplatin (0.5, 1, and 2.5 μM) were analyzed by CalcuSyn software. CI < 1 indicates synergistic effect. **(D)** Flow cytometric analysis of the control and A549 cells treated with 1.5 µM niraparib and 5 µM cisplatin, individually and in combination, labeled with PI/annexin staining. **(E)** Quantifications and statistical analyses of flow cytometric analysis of the control and A549 cells treated with 1.5 µM niraparib and 5 µM cisplatin. **(F)** Western blot analysis of cell lysates from the control and A549 cells treated with 1.5 µM niraparib and 5 µM cisplatin, individually and in combination, performed with anti-lamin B, anti-PARP1, anti-caspase-3 antibodies, and anti-β-tubulin as the loading control. The results presented as a bar graph are the means ± SD. Statistically significant differences between groups are indicated with * with a p-value ≤0.05 (*), ≤0.01 (**), ≤0.001 (***), and ≤0.0001 (****).

To investigate the potential of niraparib to augment the action of cisplatin, the interaction between niraparib and cisplatin during combined treatment in the A549 cell line was assessed by MTT assay (Figures 3A, B). Niraparib potentiated the effect of cisplatin in A549 cells in a way that 0.5, 1, and 1.5 µM of niraparib significantly reduced the IC_50_ values of cisplatin, which decreased almost 2.0-fold in comparison to that in cisplatin-only treated cells (p < 0.0001 for all niraparib concentrations). The results were then subjected to computerized synergism/antagonism CalcuSyn software analysis that showed a synergistic effect between niraparib and cisplatin with CI < 1 in all the tested combinations of drug concentrations (Figure 3C). The nature of the interaction between niraparib and cisplatin was also assessed by SynergyFinder+ online tool (Supplementary S1 SynergyFinder report). All the applied models showed positive scores, suggesting a synergistic interaction. HSA showed the highest synergy (8.74), indicating that the combination outperforms the best single agent, which is cisplatin in our case. The Loewe additivity model, the most conservative model, also showed a positive score (5.43). Subsequent flow cytometric analysis revealed a cell death-inducing activity of niraparib and cisplatin individually and in combined treatment of the A549 cell line (Figure 3D). The results of the quantification of PI/annexin V staining indicated that both drugs, niraparib and cisplatin, induce the apoptotic cell death pathway but to a different extent (Figure 3E). Annexin V staining (annexin V+, Figure 3E) indicated an intensive increase in the percentage of apoptotic cells in the A549 culture subjected to combined niraparib and cisplatin treatment (32.993%) compared to the cells treated with niraparib (7.68%) or cisplatin (22.123%) and the non-treated control cells (2.983%). In all the A549 cultures subjected to the previously listed treatments, the majority of the apoptotic cells were at an early apoptotic stage, i.e., annexin V + PI (niraparib 3.973%, cisplatin 17.220%, and niraparib + cisplatin 23.883%). The higher degree of apoptotic cells among the cisplatin-treated population than in the niraparib-treated population was expected, given the low dose of niraparib used in the experiments. Importantly, according to the quantification of annexin V+ staining, niraparib and cisplatin in combination potentiates the apoptosis rate in A549 cells compared to niraparib (p < 0.0001) or cisplatin (p < 0.0001) individual treatments, which could be attributed to their synergism. Triggering the apoptotic pathway upon treatment with niraparib, cisplatin, and their combination was confirmed by assessing the expression levels of lamin B, PARP1, and procaspase-3 in the A549 cell line by Western blotting (Figure 3F). Lamin B and PARP are well-known targets of effector caspases, whose cleavage is used as a hallmark of apoptotic cell death, while caspase-3 is the main effector caspase, which is exclusively involved in apoptotic proteolysis. Although A549 cells were treated with a low concentration of niraparib (1.5 µM), the decrease in procaspase-3 full length and lamin B protein levels indicates the involvement of apoptotic mechanisms. Treatment with niraparib did not affect the expression level of PARP1, indicating that the effect of niraparib on PARP1 activity (Figure 2B) is not related to PARP1 expression. On the other hand, as expected, cisplatin alone and in combination with niraparib markedly reduced the expression levels of lamin B, procaspase-3, and PARP proteins.

### 3.4 Different modulation of niraparib activity by DMOG and vitamin C in A549 cells

**FIGURE 4:**
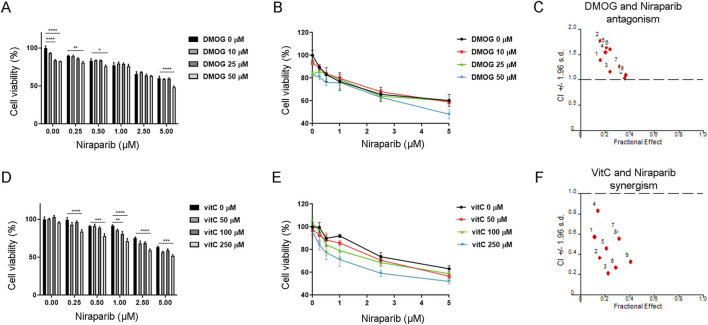
Interaction between niraparib and the modulators of TET enzymatic activity, with DMOG as the TET inhibitor or vitamin C as an activator of TET. **(A, B)** MTT viability assay of A549 cells upon combined treatment with increasing concentrations of niraparib (0 µM–5 µM) in combination with increasing concentrations of DMOG (0 µM–50 µM). **(C)** Result of the computerized synergism/antagonism CalcuSyn software analysis of combination treatment with niraparib (0.5, 1, and 2.5 μM) and DMOG (10, 25, and 50 μM). CI > 1 indicates antagonism, while CI close to 1 points to an additive effect. **(D, E)** MTT viability assay of A549 cells upon combination treatment with increasing concentrations of niraparib (0 µM–5 µM) in combination with increasing concentrations of vitamin C (0 µM–250 µM). **(F)** Result of computerized synergism/antagonism CalcuSyn software analysis of combined niraparib (0.5, 1, and 2.5 μM) and vitamin C (50, 100, and 250 μM) treatments. CI < 1 indicates a synergistic effect. The results presented as bar graphs **(A, D)** are the means ± SD. Statistically significant differences between groups are indicated with * with a p-value ≤0.05 (*), ≤0.01 (**), ≤0.001 (***), and ≤0.0001 (****).

The previous result revealed that niraparib in combination with cisplatin potentiates apoptosis and improves the responsiveness of the A549 cell line to cisplatin. Since we hypothesized that the chemosensitization effect of PARPi could involve the activation of TET-dependent hydroxymethylation and 5hmC production, we analyzed the effect of niraparib on cell viability in the presence of substances that are already shown to inhibit (DMOG) or activate (vitamin C) the TET enzymatic activity. The interaction between niraparib and DMOG, which is supposed to inhibit TET activity, was assessed by MTT assay upon combined treatment in the A549 cell line (Figures 4A, B). DMOG in the concentrations of 25 μM and 50 µM significantly reduced cell viability in the absence of niraparib (p < 0.0001 for both concentrations), although the achieved inhibition of cell growth did not exceed 20%. However, with the addition of niraparib, there is no significant reduction in cell viability for the 25 µM concentration of DMOG. In addition, combining 50 µM DMOG with 1 and 2.5 µM niraparib affects cell viability as much as use of niraparib alone, without additional reduction (Figure 4A). CalcuSyn software analysis revealed the antagonistic effect of DMOG with niraparib at 0.5, 1, and 2.5 µM concentrations (Figure 4C). Namely, a CI calculated from growth inhibition curves that reflect cytotoxicity was established to be ≥1, pointing out the antagonistic effect between DMOG, which was shown to inhibit TET activity (Tolić et al., 2019), and PARP inhibitor, which has been shown to activate TET (Tolić et al., 2022). On the contrary, vitamin C as a positive regulator of TET function was found to synergize with niraparib, which was established by MTT assay during combined treatment in the A549 cell line (Figures 4D, E). Treatments with vitamin C did not affect cell viability in the absence of niraparib (Figure 4D). Niraparib in concentrations of 0.25, 0.5, 1, 2.5, and 5 µM combined with 250 µM vitamin C produced an additional significant decrease in the cell viability compared to that with the treatment with niraparib alone (p = 0.0009 for 5 µM niraparib, p < 0.0001 for all other concentrations). The same effect regarding the marked reduction in cell viability was achieved when 1 µM niraparib was combined with 100 µM vitamin C (p = 0.0031).The synergism between niraparib at 0.5, 1, and 2.5 µM concentrations and vitamin C at 50, 100, and 250 µM concentrations was confirmed by CalcuSyn software analysis (Figure 4F), which determined CI to be <1 pointing out the synergistic effect between two drugs that have the ability to activate TET enzymes. These results suggested that the effectiveness of the niraparib and cisplatin combined treatment in the sensitization of A549 cells could be related to the potential of niraparib to modulate the expression or activity of TET enzymes.

### 3.5 The effect of niraparib and cisplatin on the expression and activity of TET enzymes in A549 cells

**FIGURE 5:**
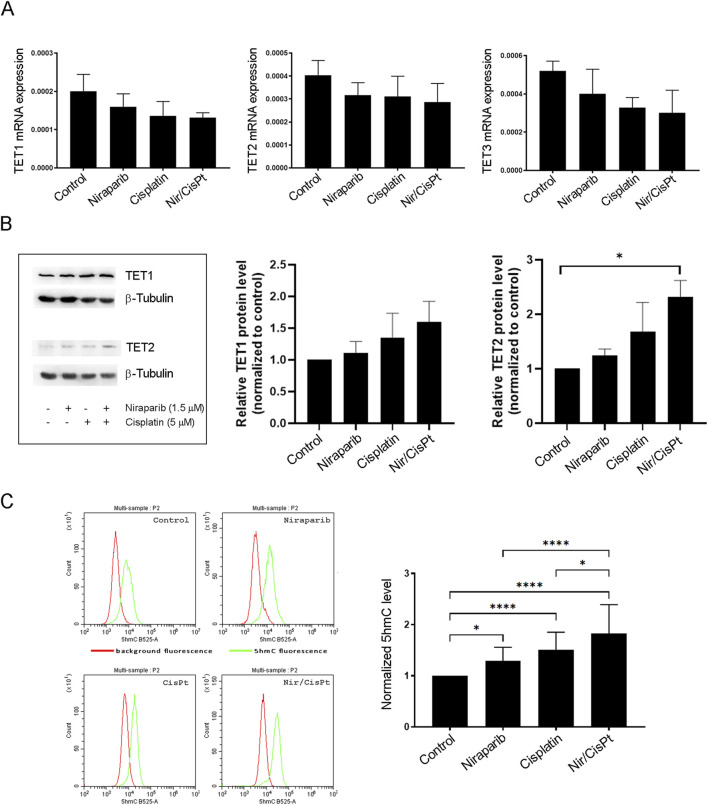
Effect of niraparib and cisplatin on the mRNA and protein expressions and activity of TET enzymes in A549 cells. **(A)** mRNA expression of TET1, TET2, and TET3 enzymes in control A549 cells and after treatment with 1.5 µM niraparib and 5 µM cisplatin, individually and in combination, obtained using real-time PCR analysis. mRNA levels are normalized to *GAPDH* mRNA expression. **(B)** Western blot analysis of cell lysates from the control and A549 cells treated with 1.5 µM niraparib and 5 µM cisplatin, individually and in combination, performed with anti-TET1 and anti-TET2 antibodies and anti-β-tubulin as a loading control. The densities of the bands were quantified and presented as the means ± SD after normalization to β-tubulin. **(C)** Flow cytometric analysis of the control and A549 cells treated with 1.5 µM niraparib and 5 µM cisplatin, individually and in combination, labeled with the anti-5hmC antibody. The results of quantification are presented as the means ± SD. Statistically significant differences between groups are indicated with * with a p-value ≤0.05 (*), ≤0.01 (**), ≤0.001 (***), and ≤0.0001 (****).

In order to assess whether niraparib and cisplatin individually and in combined treatment affect the TET abundance (TET1/2/3), the mRNA expression of TETs was analyzed by real-time PCR (Figure 5A). The mRNA expression levels for all three TET enzymes downregulated upon niraparib treatment, but with no statistical significance. A similar trend by means of decreased mRNA expression was observed for all TET enzymes upon cisplatin treatment and combined treatment with niraparib and cisplatin, but this also was without statistically significant changes (Figure 5A). The protein expression level of TET1 and TET2 enzymes was determined using Western blot analysis (Figure 5B). All treatments led to increase in the amount of TET1 and significantly increased the level of TET2 protein in niraparib and cisplatin combined treatment compared to the control (p = 0.0425). Since we postulated that niraparib would potentiate the response of A549 to cisplatin via increased hydroxymethylation that arises from TET activity, the hydroxylase activity of TET enzymes related to 5hmC production was assessed in A549 cells treated with niraparib and cisplatin individually and in combination. The flow cytometric analysis of A549 cells labeled with anti-5hmC antibody was performed and demonstrated that the 5hmC level significantly increased upon treatment with niraparib (p = 0.0406), cisplatin (p < 0.0001), and their combined treatment (p < 0.0001) compared to untreated cells. Moreover, the level of 5hmC upon joint treatment significantly exceeds the level detected upon individual niraparib (p < 0.0001) and cisplatin (p = 0.0218) treatments (Figure 5C). Therefore, increased TET activity and restoration of the 5hmC level could be the cause of the observed synergistic effect and increased sensitivity of A549 cells to cisplatin when treated in combination with the PARPi niraparib.

## 4 Discussion

Recent research on the use of PARP inhibitors—clinically proven chemosensitizers for ovarian and breast cancers with BRCA mutations—has been expanding to include lung cancer treatment. This research suggests that lung cancer cells may exhibit varying susceptibilities to the synergistic effects of PARP inhibitors and cisplatin in chemosensitization (Ji et al., 2020; Stolzenburg et al., 2022; Diéras et al., 2020). Considering the heterogeneity among NSCLC cells susceptible for chemosensitization with PARPi (Stolzenburg et al., 2022) and that deficiency in DNA damage repair genes were not required for PARPi to act in NSCLC (Khaddour et al., 2022), the identification of new molecular targets for PARPi in lung cancer is a promising approach to select a novel parameter for efficient stratification of patients who will most likely benefit from treatment. The inhibitory mechanism of PARPi involves the following: (i) the catalytic inhibition of PARP and (ii) PARP trapping, which is assumed to be more efficient in PARP inhibition. The PARPi with the highest PARP trapping activity, talazoparib and niraparib, were found to be most effective in increasing the cytotoxicity of cisplatin in bladder cancer cells (Bhattacharjee et al., 2022). Since niraparib was used in our previous studies, which revealed that the inhibition of PARP1-dependent PARylation induces TET-mediated DNA hydroxymethylation (Tolić et al., 2022), we additionally used niraparib in this study for the improvement in sensitivity to cisplatin in NSCLC cells. In this study, it was revealed that niraparib-dependent stimulation of the activity of the TET enzyme and subsequent restoration of the 5hmC level could be the pathway for increasing sensitivity to cisplatin in A549 cancer cells that exhibited the chemoresistant phenotype.

Numerous studies have shown that the use of a demethylating agent DNMT inhibitor 5-aza, 2′-deoxyCytidine (5 AZA CdR or Decitabine) in combination with chemotherapeutics could recover sensitivity to chemotherapeutic agents such as cisplatin (Lima et al., 2022; Wang et al., 2023; Camero et al., 2021). The hypomethylating 5 AZA CdR was found to sensitize lung, melanoma, hepatoma, breast, prostate, and pancreatic cancers with diverse pathways of synergy, with the assumption that chemosensitization mostly results from stimulation of apoptosis (Mirza et al., 2010). Nowadays, the disturbance of active demethylation pathways such as deficiency in TET activity and 5hmC loss is considered to be associated with chemotherapy resistance and tumor progression. Therefore, apart from the genetic profile and mutation status that have been used to predict the response to targeted therapy (Dinić et al., 2024), the recent evidence suggests the critical role of the patient’s epigenetic landscape in influencing the therapeutic outcomes. Emerging evidence indicates that global reduction of 5hmC can be correlated with chemotherapy resistance, implying that the tumor or its subtype can be categorized based on 5hmC levels as a potential marker of chemoresistance (Kharat and Sharan, 2020). Accordingly, our results revealed that among examined cell lines—human, sensitive NSCLC cells NCI-H460, resistant NCI-H460/R cells, lung adenocarcinoma cells A549, and normal embryonic lung fibroblasts MRC-5—the A549 cell line, which exhibited the most pronounced resistance to cisplatin, was characterized by the lowest genome level of 5hmC. It was recently found that TET-related 5hmC deficiency plays an essential role in the development of chemotherapy resistance in hepatocellular carcinoma, suggesting that the loss of 5hmC could be considered a valuable parameter for the prediction of the response to chemotherapy and a new therapeutic target for chemotherapy-resistant HCC patients (Guo et al., 2023). Our previous results, which firmly proved that the inhibition of PARP-dependent PARylation (the covalent attachment of poly-ADP-ribose units to acceptor proteins) activates TET enzyme and hydroxy/demethylation in NIH3T3 cells (Tolić et al., 2022), provided a rationale for the hypothesis that DNA hydroxy/demethylation boosted by PARP inhibition could restore tumor sensitivity via TET activation. We showed that cisplatin in combination with a low dose of niraparib showed a synergistic growth-suppressive effect on the A549 cell line, thus pointing to cisplatin-resistance reversal. The reduction in cisplatin IC_50_ values appears similar across niraparib doses, indicating that even the lowest tested dose (0.5 µM) of niraparib is sufficient to synergistically augment cisplatin’s efficacy in A549 cells. This is of importance since PARPi can be used as effective chemosensitizers in combined treatment at much lower concentrations (de Haan et al., 2018).

The growth suppression mechanism induced by cisplatin or/and niraparib was found to involve apoptosis with activated caspase-dependent signaling pathways in A549 lung cancer cells. Since apoptosis reduction is one of the critical features of chemotherapy-resistant tumor cells, it is expected that chemosensitization with demethylating agents will stimulate the apoptosis pathway (Mirza et al., 2010). For example, pretreatment of platinum-resistant ovarian cancer, which was characterized with low levels of 5hmC, with hypomethylating DNMT inhibitor resulted in increased 5hmC level and apoptotic signaling, revealed by the cleaved caspase-3 level (Bisht et al., 2022; Tucker et al., 2018). Although the effects of combined treatment on apoptosis could be mostly attributed to cisplatin, the combination treatment with the low concentration of niraparib (1.5 µM) can deliver more intense effects. In fact, it is necessary for an agent that sensitizes cells to cisplatin to be applied at lower concentrations with little, if any, toxicity for non-tumor cells (Yu et al., 2018).

Our reported data that the cellular response to niraparib is mediated at least in part by induced TET activity (Tolić et al., 2022) was confirmed in this study by the result showing that niraparib-dependent inhibition of A549 cell growth is affected in a striking synergistic manner by vitamin C, an activator of TETs. In addition, the antagonistic effect between niraparib and DMOG, a nonspecific inhibitor of 2-OG-dependent dioxygenase that inhibits TET1, TET2, and TET3 (Zhang et al., 2019), was detected in A549 cell viability. The enhancement in TET activity by vitamin C has been extensively studied. We previously established that the expression of gene coding for chemokine CXCL12 increased in the presence of vitamin C and decreased upon DMOG treatment in PARP^−/−^ cells, pointing out the involvement of TET activity in gene regulation in the absence of PARP (Tolić et al., 2019). In accordance, vitamin C was found to enhance TET2 activity and recruitment to promoter regions of chemokine, resulting in its increased expression in (B16-OVA) melanoma cells (Yue and Rao, 2020). It was reported that treatment with 5 AZA demethylating agents reversed cisplatin resistance in NSCLC by the re-expression of epigenetically silenced genes involved in development of chemoresistance (Zhang et al., 2014). Aside from changing the cellular programs related to cisplatin resistance (by demethylation/re-expression of corresponding genes), the DNA demethylation pathway could act in chemopotentiation independently of gene activation. The augmented TET activity encompassing the concurrent 5hmC increase can cause replication fork (RF) degradation, which ultimately results in DNA breakage and cell death, thus reverting the chemoresistance (by rendering the stalled RF susceptible/vulnerable to degradation) (Kharat et al., 2020). Considering that PARPi could block the restarting of the stalled RF causing sustained DNA damage and consequently potentiating the cytotoxicity of cisplatin, the additional layer of PARPi effects may include activation of the TET enzyme and accumulation of 5hmC across the genome, which contributes to genome instability (which is a well-known effect of hypomethylation) (Srivastava and Lodhi, 2022; Jung et al., 2019). Our results showed that niraparib as a TET activator did not influence the expression of mRNA for TET proteins in a statistically significant manner as neither cisplatin nor their combined treatment did. The effect of niraparib on TET hydroxylase activity in terms of 5hmC production resulted rather from TETs’ catalytic activities, which was revealed in our previous studies (Tolić et al., 2022), than from altered gene expression of TET enzymes. However, the protein level of TET2 statistically increased upon co-treatment with cisplatin and niraparib, which is in accordance with the elevation in the 5hmC level and increased apoptosis upon combination treatment. The combined effect of niraparib and cisplatin on TET2 protein expression and increased 5hmC production could be achieved by the induction of TET activity by niraparib and by induction of DNA damage with cisplatin since the specific enrichment of 5hmC as a response to DNA damage was documented (Kantidze and Razin, 2017). The relevance of this result lies in the fact that TET2 has been emphasized as an important player in cisplatin sensitivity, where its loss mediates cisplatin resistance (Kharat et al., 2020).

Overall, our results indicated that boosting 5hmC levels could provide a molecular basis for the cellular response to overcome resistance to cisplatin in NSCLC cells, which can be achieved by the stimulating effects of PARPi on TET enzymes. However, possibility that PARPi, acting as a trapping agent, induces TET activity via replication stress-mediated DNA damage could not be ruled out (Kharat et al., 2020; Rath et al., 2024). Thus, the extent to which the induction of DNA damage by PARP trapping and activation of TETs by PARP inhibition contributes to TET-related increase in 5hmC production is yet unknown, and it has to be demonstrated in future research. Hereof, deeper molecular insights should be gained to define the epigenetic pattern that could be targeted to potentiate chemosensitization and overcoming drug resistance. The results of this study indicate that the determination of the 5hmC level could provide a convenient method of screening patients who might benefit from PARPi-based induction of TET enzymes in terms of tumor resistance to cisplatin. Potential clinical studies should include the examination of a patient’s 5hmC level for assessing its prognostic value and susceptibility to therapy that would affect TET enzyme levels, increase the 5hmC level, and reverse chemoresistance.

## Data Availability

The original data presented in the study are included in the article/Supplementary Material, further inquiries can be directed to the corresponding authors.
