# Supplementary material for: PARP inhibition increases sensitivity to cisplatin in non-small-cell lung carcinoma via the induction of TET-dependent hydroxymethylation
Source: Front Cell Dev Biol. 2025 Oct 24;13:1677261. doi: 10.3389/fcell.2025.1677261 (PMC12592190; doi:10.3389/fcell.2025.1677261)
Supplement: Supplementary file 1 [file DataSheet1.pdf]

SynergyFinder+ Report

https://synergyfinder.org/ http://synergyfinder.ai/ www.synergyfinderplus.org
https://tangsoftwarelab.shinyapps.io/synergyfinder

2025-08-21

```
## Correct Baseline Setting:  non
```

Data tables

Table 1: Drug Combiantion Meta Data

Block ID	$Drug_1$	$Drug_2$	Conc $Unit_1$	Conc $Unit_2$
1	Nirapparib	Cisplatin	uM	uM

Table 2: Synergy Score Summary Table

Block ID	ZIP	Loewe	HSA	Bliss
1	5.92	5.43	8.74	6.57

Reference

For use of the SynergyFinder+ R package or the web application:

[1] Zheng, S.; Wang, W.; Aldahdooh, J.; Malyutina, A.; Shadbahr, T.; Tanoli, Z.; Passia, A.; Tang, J. SynergyFinder Plus: Toward Better Interpretation and Annotation of Drug Combination Screening Datasets. Genomics, Proteomics & Bioinformatics 2022, 20 (3), 587-596. doi:10.1016/j.gpb.2022.01.004.

For use of ZIP synergy scoring:

[2] Yadav, B.; Wennerberg, K.; Aittokallio, T.; Tang, J. Searching for Drug Synergy in Complex Dose-Response Landscapes Using an Interaction Potency Model. Comput Struct Biotechnol J 2015, 13, 504–513. doi:10.1016/j.csbj.2015.09.001

For how to harmonize the different synergy scoring methods:

[3] Tang, J.; Wennerberg, K.; Aittokallio, T. What Is Synergy? The Saariselkä Agreement Revisited. Front Pharmacol 2015, 6, 181. doi:10.3389/fphar.2015.00181

For general ideas of drug combination therapies:

[4] Tang, J. Informatics Approaches for Predicting, Understanding, and Testing Cancer Drug Combinations. Methods Mol Biol 2017, 1636, 485–506. doi:10.1007/978-1-4939-7154-1_30

For retrieving the most comprehensive drug combination data resources and their sensitivity and synergy results by SynergyFinder, please go to DrugComb :

[5] Zheng, S.; Aldahdooh, J.; Shadbahr, T.; Wang, Y.; Aldahdooh, D.; Bao, J.; Wang, W.; Jing, T. DrugComb update: a more comprehensive drug sensitivity data repository and analysis portal. Nucleic Acids Research 2021, 49 (w1), w174-w184. doi:10.1093/nar/gkab438

[6] Zagidullin, B.; Aldahdooh, J.; Zheng, S.; Wang, W.; Wang, Y.; Saad, J.; Malyutina, A.; Jafari, M.; Tanoli, Z.; Pessia, A.; Tang, J. DrugComb: An Integrative Cancer Drug Combination Data Portal. Nucleic Acids Res 2019, 47 (W1), W43–W51. doi:10.1093/nar/gkz337

For use of combination sensitivity score:

[7] Malyutina, A.; Majumder, M. M.; Wang, W.; Pessia, A.; Heckman, C. A.; Tang, J. Drug Combination Sensitivity Scoring Facilitates the Discovery of Synergistic and Efficacious Drug Combinations in Cancer. PLOS Computational Biology 2019, 15 (5), e1006752. doi:10.1371/journal.pcbi.1006752

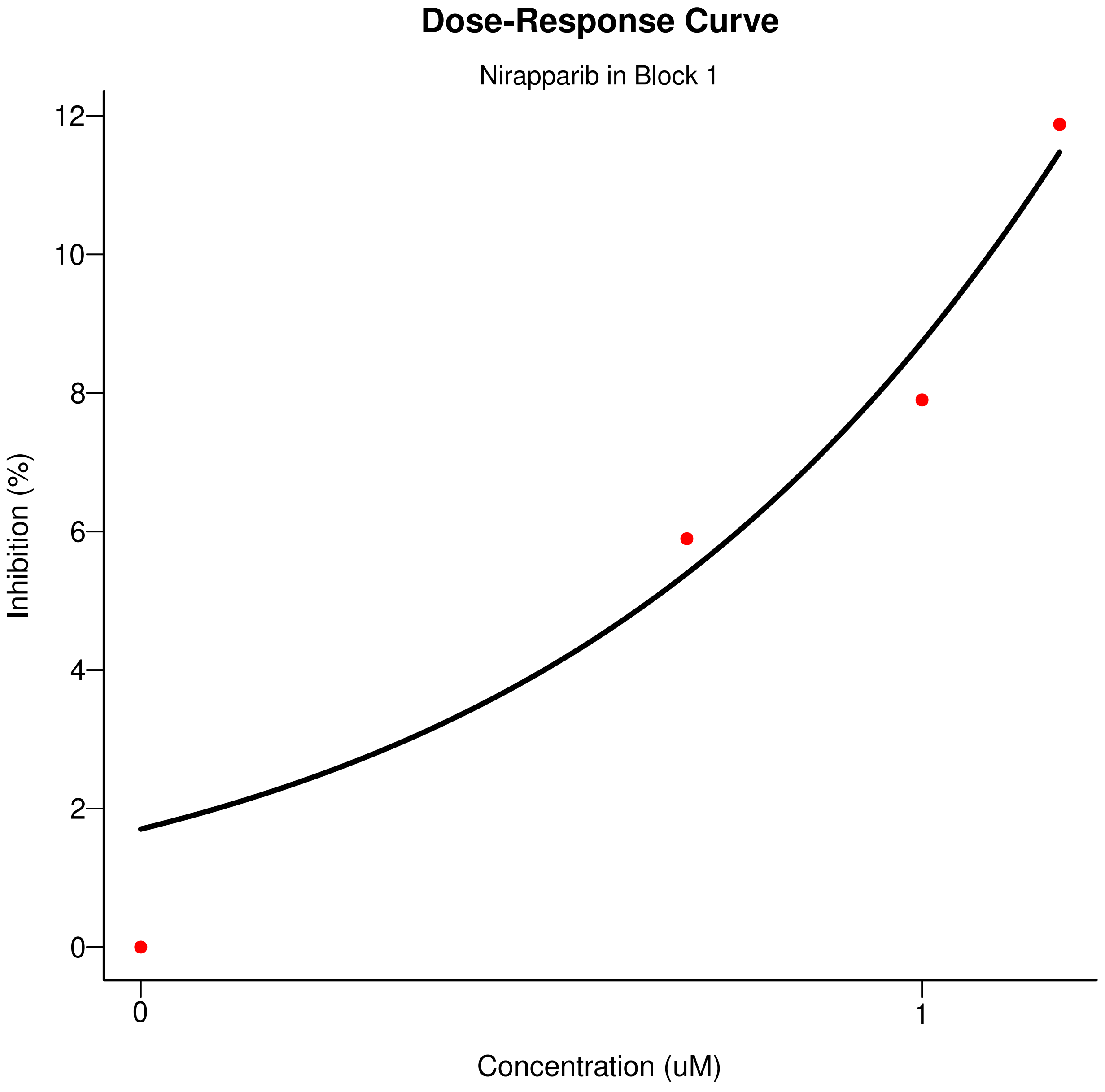

Dose-Response Curve
Nirapparib in Block 1
Inhibition (%)
0
2
4
6
8
10
12
0
1
Concentration (uM)

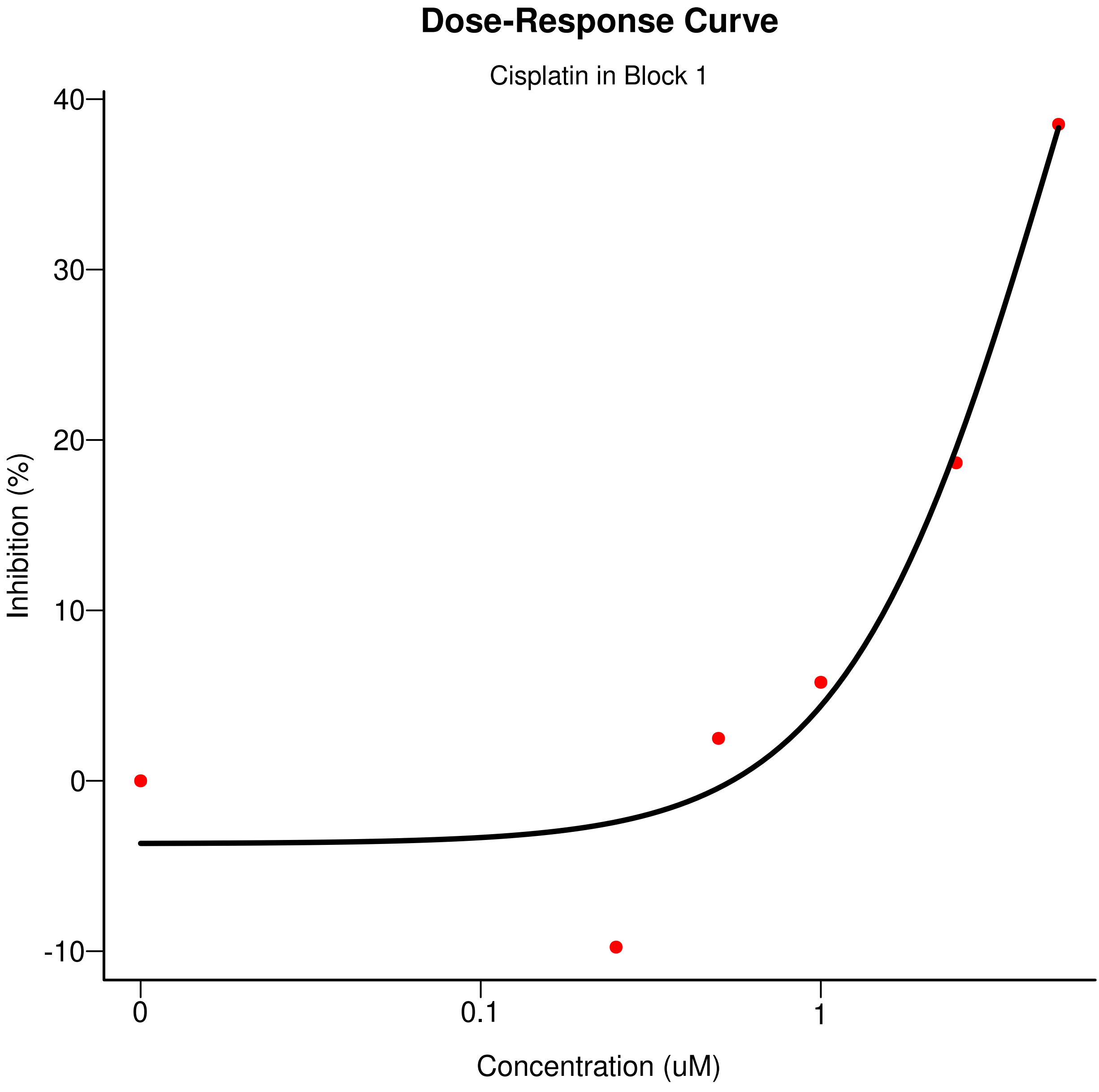

Dose-Response Curve
Cisplatin in Block 1
40
30
20
10
0
-10
Inhibition (%)
0
0.1
1
Concentration (uM)

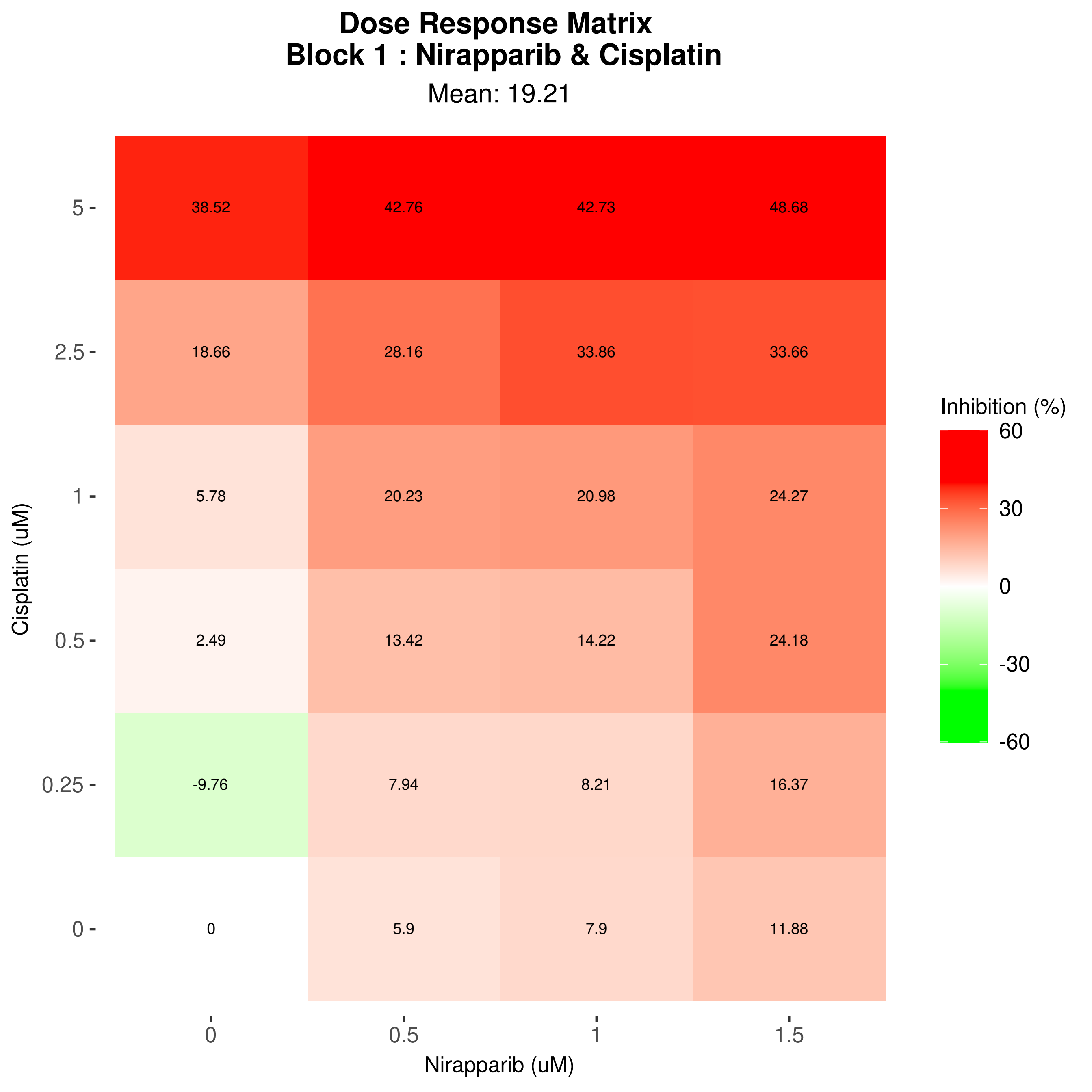
Dose Response Matrix
Block 1 : Nirapparib & Cisplatin
Mean: 19.21
5
2.5
1
0.5
0.25
0
38.52
42.76
42.73
48.68
18.66
28.16
33.86
33.66
5.78
20.23
20.98
24.27
2.49
13.42
14.22
24.18
-9.76
7.94
8.21
16.37
0
5.9
7.9
11.88
0
0.5
1
1.5
Nirapparib (uM)
Cisplatin (uM)
Inhibition (%)
60
30
0
-30
-60

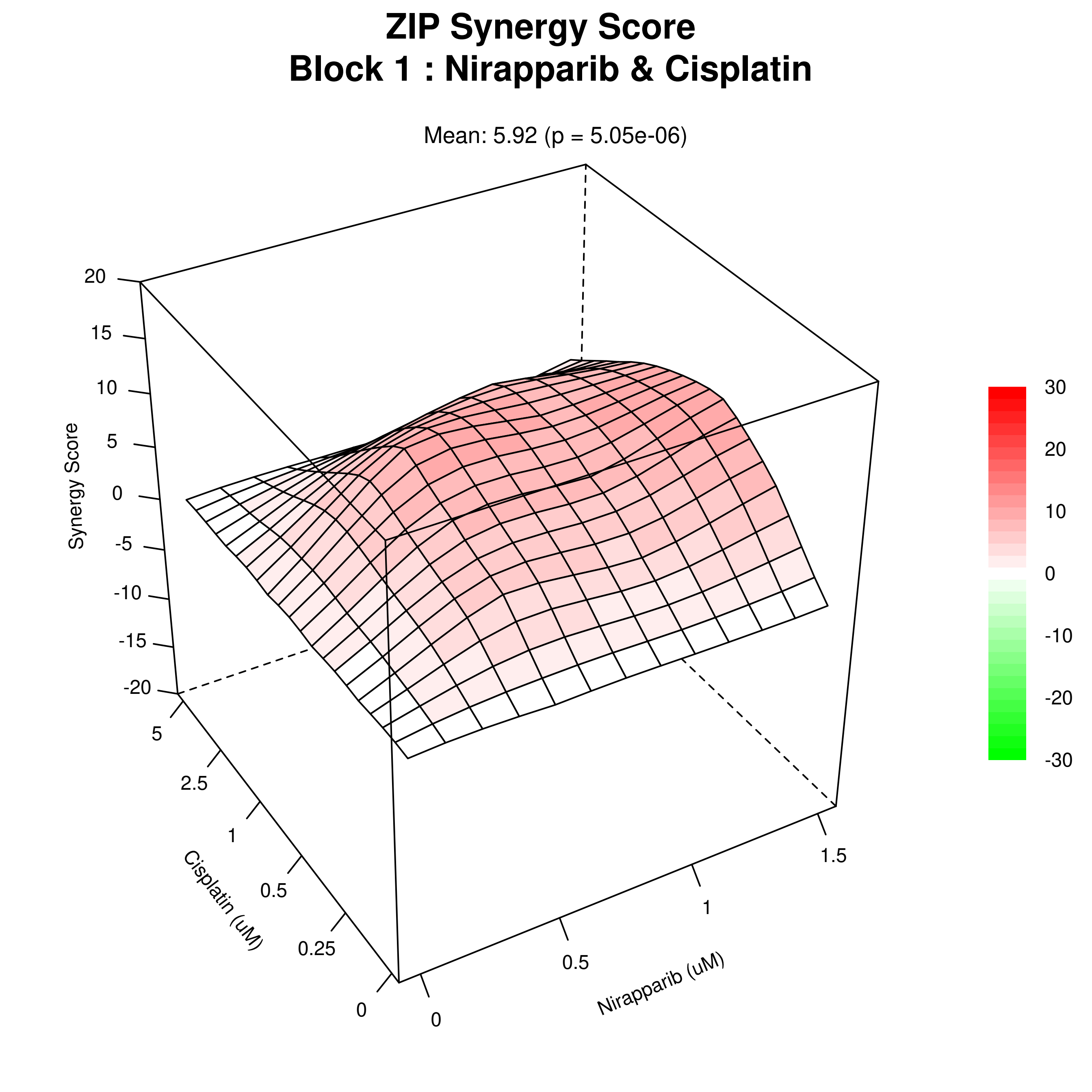
ZIP Synergy Score
Block 1 : Nirapparib & Cisplatin
Mean: 5.92 (p = 5.05e-06)
Synergy Score
20
15
10
5
0
-5
-10
-15
-20
Cisplatin (uM)
5
2.5
1
0.5
0.25
0
Nirapparib (uM)
0
0.5
1
1.5
30
20
10
0
-10
-20
-30

Loewe Synergy Score
Block 1 : Nirapparib & Cisplatin

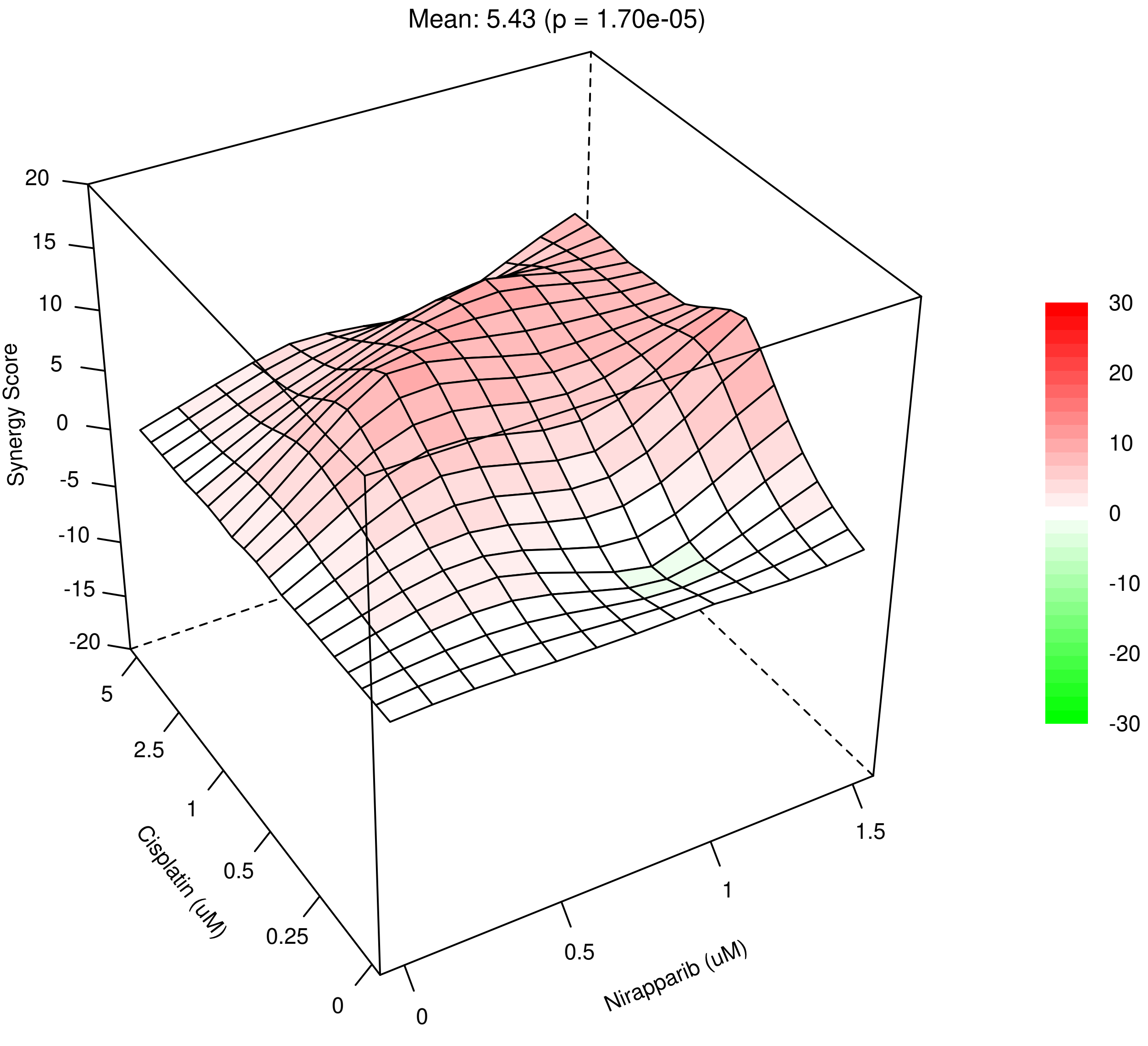

Bliss Synergy Score
Block 1 : Nirapparib & Cisplatin

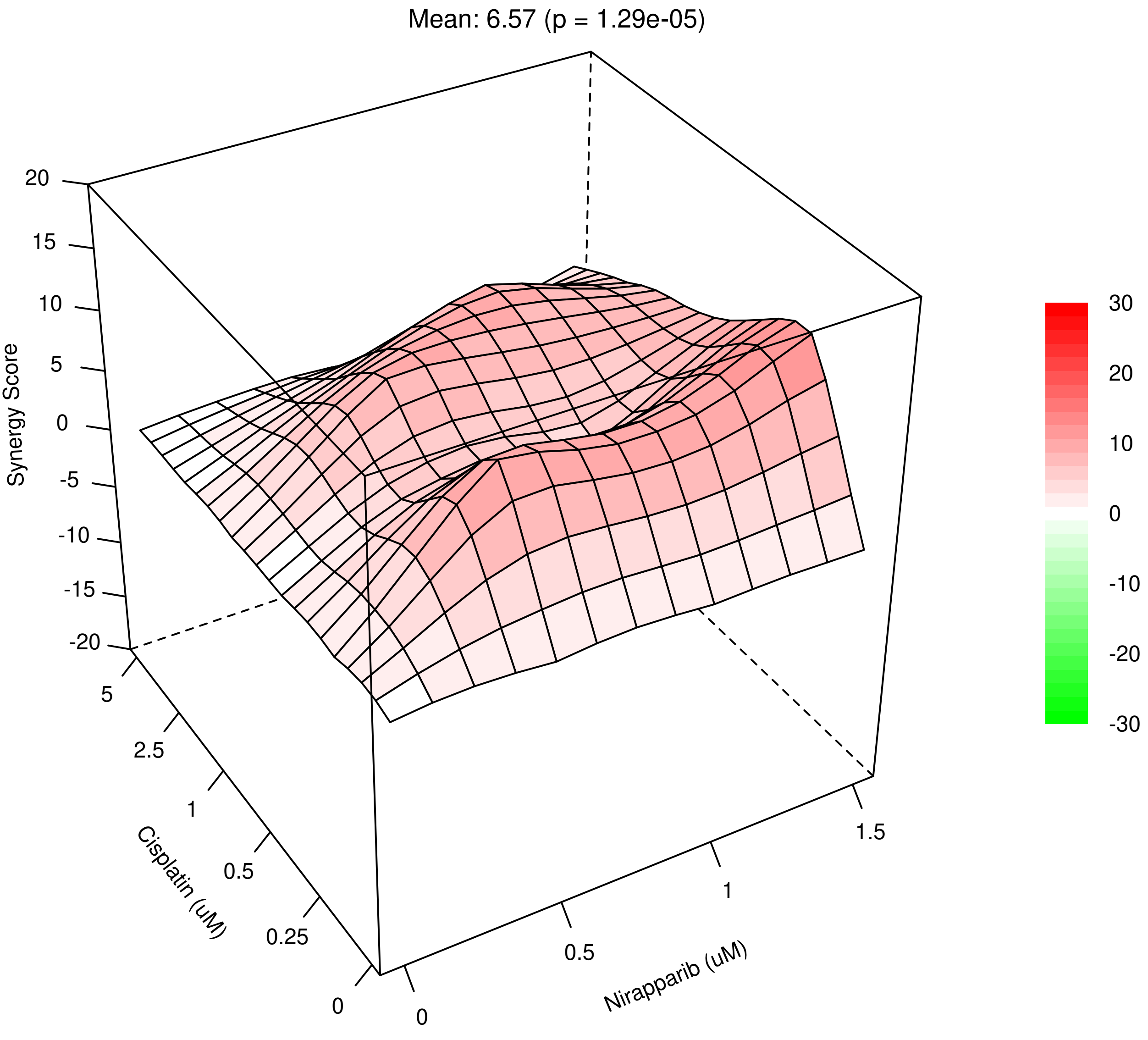

HSA Synergy Score
Block 1 : Nirapparib & Cisplatin

Mean: 8.74 (p = 7.91e-06)

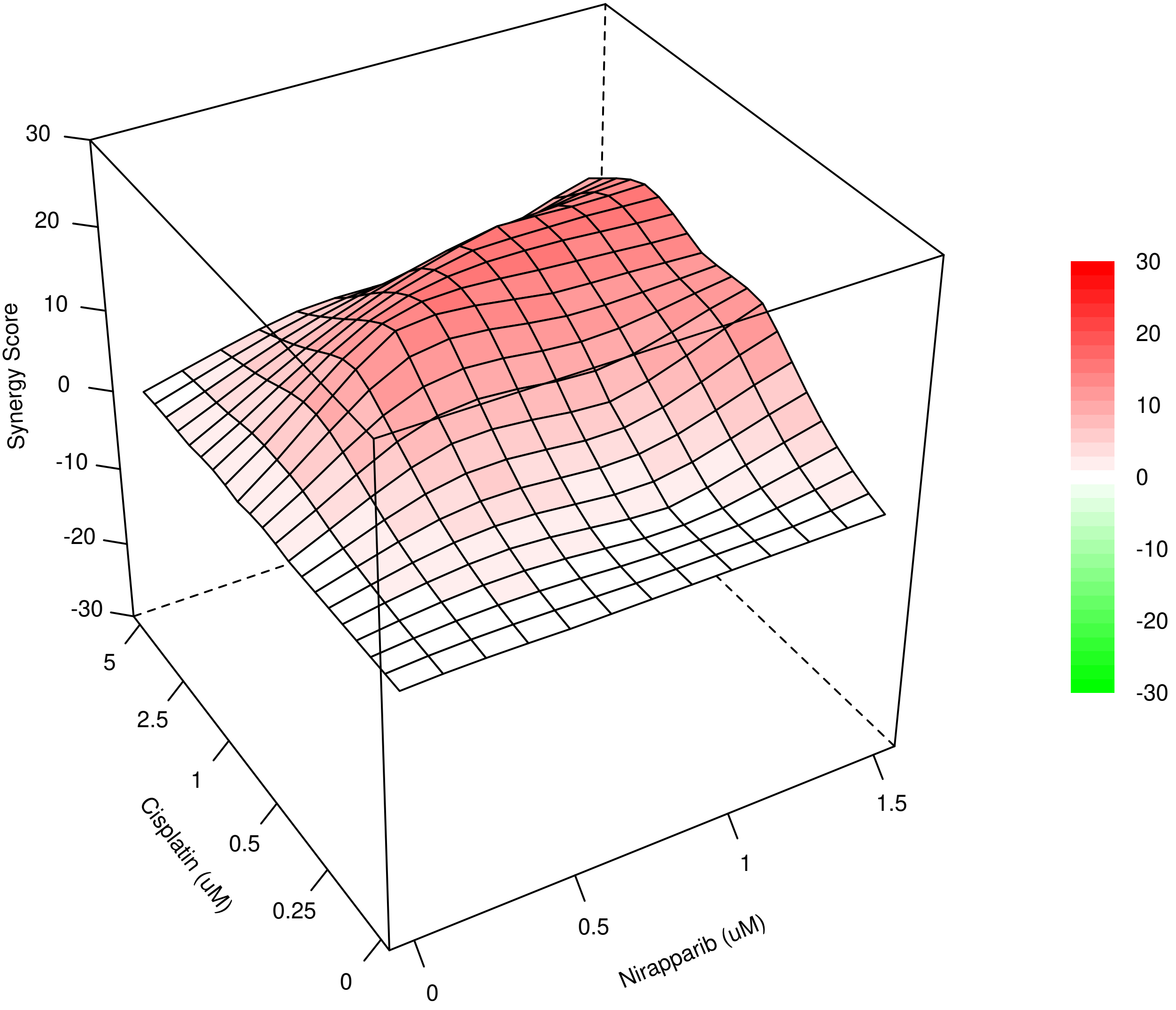

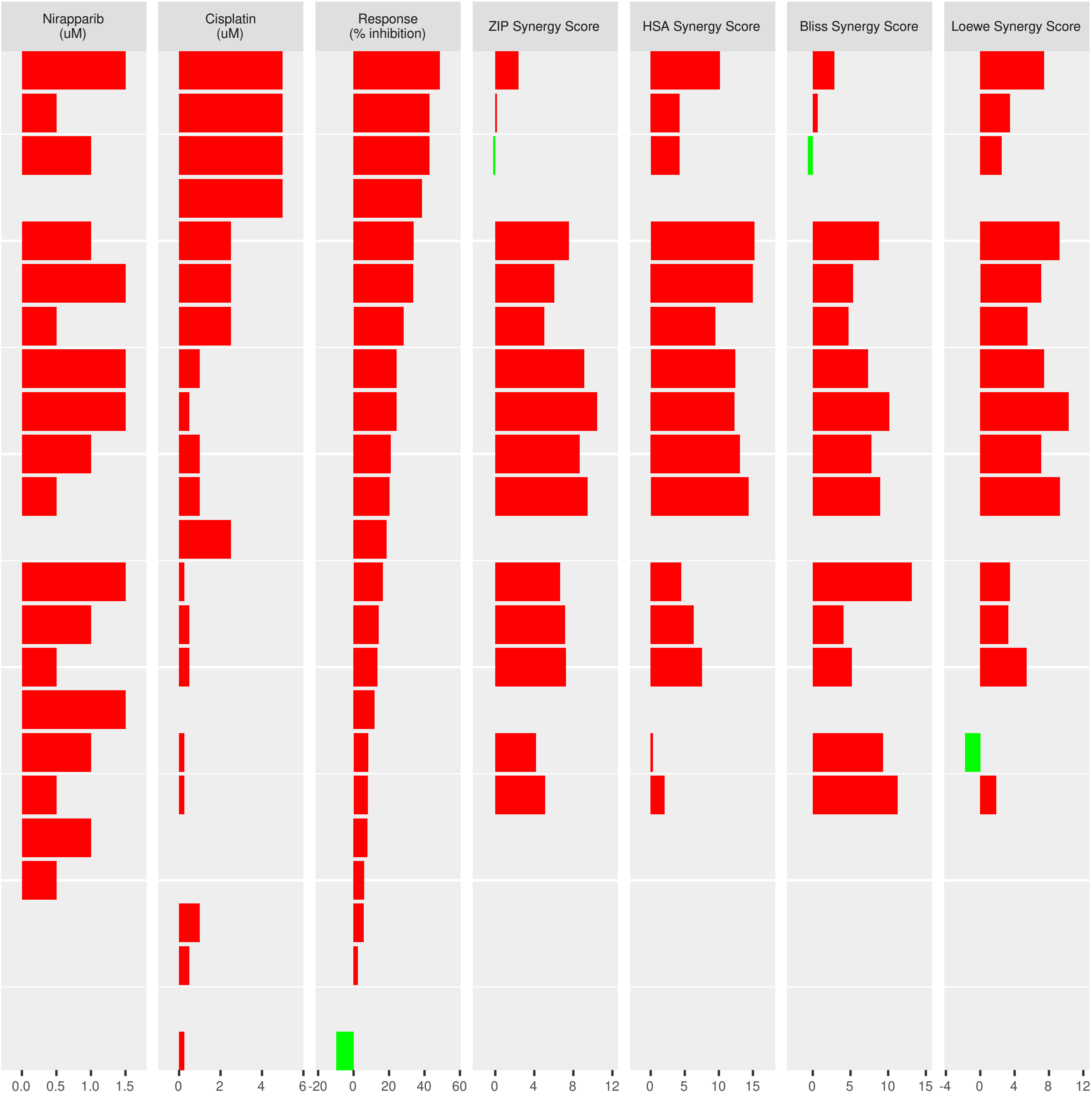
Nirapparib (uM)
Cisplatin (uM)
Response (% inhibition)
ZIP Synergy Score
HSA Synergy Score
Bliss Synergy Score
Loewe Synergy Score
0.0 0.5 1.0 1.5
0 2 4 6
-20 0 20 40 60
0 4 8 12
0 5 10 15
0 5 10 15
-4 0 4 8 12
